# Supplementary material for: Impact of the COVID-19 Pandemic on Mental Health among Patients with Chronic Ocular Conditions
Source: Vision (Basel). 2023 Jul 11;7(3):49. doi: 10.3390/vision7030049 (PMC10366822; doi:10.3390/vision7030049)
Supplement: Supplementary file 1 [file vision-07-00049-s001.zip › vision-2442736-supplementary.pdf]

vision

MDPI

Supplementary Materials: Impact of the COVID-19 Pandemic on Mental Health among Patients with Chronic Ocular Conditions

Soumaya Bouhout, Mélanie Hébert, Weronika Jakubowska, Laurence Jaworski, Ellen E. Freeman and Marie-Josée Aubin

Table S1. Baseline characteristics of the cohort by principal disease category.

Characteristic	Glaucoma, n=31	AMD, n=34	PDR, n=37	Chronic uveitis, n=43	p-value
Age	73 [64, 76]	77 [73, 84]	66 [58, 72]	59 [45, 67]	<0.001
Female sex	18, 58%	22, 65%	9, 24%	26, 35%	<0.001
Ethnicity					
Caucasian	18, 58%	25, 74%	21, 57%	24, 56%	0.38
Asian	1, 3%	0, 0%	2, 5%	3, 7%	0.47
Black	0, 0%	0, 0%	2, 5%	0, 0%	0.12
Afro-caribbean	1, 3%	0, 0%	0, 0%	0, 0%	0.30
Native Indian	0, 0%	0, 0%	0, 0%	0, 0%	NA
Hispanic	1, 3%	1, 3%	0, 0%	1, 2%	0.77
Unknown	9, 29%	8, 24%	11, 30%	14, 33%	0.86
Other	1, 3%	0, 0%	1, 3%	2, 5%	0.67
Visual acuity (VA), *logMAR*					
Right eye	0.14 [0.00, 0.33]	0.30 [0.18, 0.88]	0.30 [0.10, 0.35]	0.10 [0.00, 0.30]	<0.001
Left eye	0.10 [0.00, 0.25]	0.42 [0.17, 1.55]	0.30 [0.10, 0.57]	0.10 [0.00, 0.33]	<0.001
Best VA eye	0.00 [0.00, 0.14]	0.18 [0.10, 0.31]	0.10 [0.04, 0.30]	0.01 [0.00, 0.18]	<0.001
Worst VA eye	0.18 [0.10, 0.60]	0.79 [0.32, 2.30]	0.40 [0.24, 0.85]	0.25 [0.00, 0.55]	<0.001
Intraocular pressure, *mmHg*					
Right eye	16 [13, 18]	15 [10, 20]	16 [12, 17]	16 [15, 19]	0.64
Left eye	14 [10, 17]	15 [12, 18]	15 [12, 18]	16 [15, 20]	0.15
Systemic medical conditions					
No medical condition	5, 16%	4, 12%	0, 0%	5, 12%	0.12
Interstitial lung disease	0, 0%	1, 3%	0, 0%	0, 0%	0.35
Chronic obstructive pulmonary disease or asthma	2, 7%	3, 9%	2, 5%	3, 7%	0.95
Diabetes	6, 19%	5, 15%	37, 100%	4, 9%	<0.001
Morbid obesity, *body mass index >40*	0, 0%	0, 0%	2, 5%	0, 0%	0.12
Hypertension	11, 36%	15, 44%	26, 70%	11, 26%	<0.001
Dyslipidemia	7, 23%	14, 41%	20, 54%	8, 19%	0.003
Cardiovascular disease (CAD, CHF)	2, 7%	7, 21%	4, 11%	2, 5%	0.12
Pulmonary hypertension	0, 0%	0, 0%	0, 0%	0, 0%	NA
Chronic kidney disease or end-stage renal disease	1, 3%	1, 3%	5, 14%	0, 0%	0.03
Cancer	2, 7%	5, 15%	2, 5%	0, 0%	0.07
Organ transplant recipient	0, 0%	0, 0%	0, 0%	0, 0%	NA
Immunodeficiency	0, 0%	0, 0%	0, 0%	0, 0%	NA
Inflammatory bowel disease	1, 3%	0, 0%	0, 0%	5, 12%	0.03
Liver disease	0, 0%	2, 6%	0, 0%	1, 2%	0.27
Chronic neurological or neuromuscular disease	1, 3%	1, 3%	0, 0%	3, 7%	0.40
Trisomy 21	0, 0%	0, 0%	0, 0%	0, 0%	NA
Psychiatric condition (e.g., schizophrenia, BP)	2, 7%	0, 0%	2, 5%	2, 5%	0.56
Rheumatologic disease	2, 7%	2, 6%	1, 3%	7, 16%	0.14
Unknown	5, 16%	1, 3%	0, 0%	14, 33%	<0.001
Other	0, 0%	4, 12%	7, 19%	3, 7%	0.06

BP = bipolar disorder; CAD = coronary artery disease; CHF = congestive heart failure.

Table S2. National Eye Institute Visual Function Questionnaire-25 subscale answers by principal disease category.

Characteristic	Glaucoma, n=31	AMD, n=34	PDR, n=37	Chronic uveitis, n=43	p-value
General Health	53 [48, 75]	58 [50, 65]	60 [43, 71]	60 [50, 75]	0.62
General Vision	60 [55, 78]	65 [49, 76]	70 [55, 80]	75 [50, 80]	0.26
Ocular Pain	88 [75, 100]	100 [72, 100]	88 [75, 100]	88 [75, 100]	0.77
Near Activities	83 [58, 96]	65 [38, 88]	83 [58, 96]	88 [67, 100]	0.02
Distance Activities	92 [83, 100]	83 [69, 92]	96 [78, 100]	96 [75, 100]	0.05
Vision Specific:					
Social Functioning	100 [100, 100]	100 [92, 100]	100 [92, 100]	100 [100, 100]	0.65
Mental Health	80 [50, 95]	61 [45, 81]	80 [53, 95]	90 [69, 94]	0.04
Role Difficulties	88 [69, 100]	78 [44, 95]	94 [63, 100]	94 [69, 100]	0.15
Dependency	100 [75, 100]	94 [50, 100]	100 [66, 100]	100 [81, 100]	0.63
Driving	79 [75, 100]	67 [50, 92]	96 [83, 100]	83 [67, 100]	0.03
Color Vision	100 [100, 100]	100 [100, 100]	100 [100, 100]	100 [100, 100]	0.75
Peripheral Vision	100 [50, 100]	100 [75, 100]	100 [75, 100]	100 [100, 100]	0.09
Composite Score	82 [68, 91]	76 [64, 85]	86 [70, 92]	87 [79, 92]	0.04
